# Optimizing wheat productivity through integrated management of irrigation, nutrition, and organic amendments

**DOI:** 10.1186/s12870-024-05213-2

**Published:** 2024-06-14

**Authors:** A. S. Farouk, Ahmed M. Abdelghany, A. A. Shehab, Sh. E. Alwakel, Khaled M. Makled, Eman Naif, Honglei Ren, Sobhi F. Lamlom

**Affiliations:** 1 Agronomy Department, Faculty of Agriculture, Al-Azhar University, Cairo, Egypt; 2 Crop Science Department, Faculty of Agriculture, Damanhour University, Damanhour, Egypt; 3 Heilongjiang Academy of Agricultural Sciences, Soybean Research Institute, Harbin, 150086 China; 4 Plant Production Department, Faculty of Agriculture Saba Basha, Alexandria University, Alexandria, 21531 Egypt

**Keywords:** Potassium bicarbonate, Composting, Drip irrigation, Sustainable agriculture, Crop Productivity

## Abstract

Enhancing wheat productivity by implementing a comprehensive approach that combines irrigation, nutrition, and organic amendments shows potential for collectively enhancing crop performance. This study examined the individual and combined effects of using irrigation systems (IS), foliar potassium bicarbonate (PBR) application, and compost application methods (CM) on nine traits related to the growth, physiology, and yield of the Giza-171 wheat cultivar. Analysis of variance revealed significant (*P* ≤ 0.05) main effects of IS, PBR, and CM on wheat growth, physiology, and yield traits over the two growing seasons of the study. Drip irrigation resulted in a 16% increase in plant height, leaf area index, crop growth rate, yield components, and grain yield compared to spray irrigation. Additionally, the application of foliar PBR at a concentration of 0.08 g/L boosted these parameters by up to 22% compared to the control. Furthermore, the application of compost using the role method resulted in enhanced wheat performance compared to the treatment including mix application. Importantly, the combined analysis revealed that the three-way interaction between the three factors had a significant effect (*P* ≤ 0.05) on all the studied traits, with drip irrigation at 0.08 g PBR rate and role compost application method (referred as Drip_0.08g_Role) resulting in the best performance across all traits, while sprinkle irrigation without PBR and conventional mixed compost method (referred as sprinkle_CK_Mix) produced the poorest results. This highlights the potential to synergistically improve wheat performance through optimized agronomic inputs.

## Introduction

Bread wheat (*Triticum aestivum* L.), is the most important grain worldwide, covering a greater portion of the Earth’s surface than any other food crop [1]. It serves as a fundamental staple crop worldwide [2]. The yield of bread wheat is subject to the impacts of global climate change [3] and is susceptible to a range of biotic and abiotic stresses that affect its productivity [2]. For more than 7000 years, wheat has served as Egypt’s main winter crop and is regarded as a strategically significant commodity [4, 5]. Although it accounts for about 33% of daily caloric intake, it fails to cover 40 to 50% of Egypt’s yearly domestic demand. This highlights the importance of increasing field output to meet consumption needs [5]. Given that enhancing wheat production is a critical national priority, numerous researchers have dedicated their efforts to both increasing wheat productivity per unit area and expanding the cultivated land. This endeavor seeks to narrow the gap between Egyptian demand and consumption.

In Egypt, the agricultural sector accounts for over 83% of the total freshwater resources. The country has a total of 9.7 million cultivated areas, with around 6.5 million of these areas located in the Delta region. These areas primarily rely on surface irrigation, while the remaining 3.2 million areas are situated in newly reclaimed soils that benefit from pressurized irrigation techniques such as sprinkler, pivot, and drip irrigation [6]. Three primary irrigation systems are commonly employed: surface irrigation, sprinkler irrigation, and drip irrigation. Drip irrigation stands out as exceptionally efficient since it directly wets only the immediate root zone of each plant. This system also enables precise application of water-soluble fertilizers and other agricultural chemicals. Drip irrigation has been reported to yield significant benefits, including up to a 100% increase in crop yields, water savings ranging from 40 to 80%, and reductions in the use of fertilizers, pesticides, and labor when compared to conventional irrigation methods [7]. Various strategies, including advancements in irrigation technologies and improved irrigation scheduling, can be adopted to maximize the effective utilization of limited water resources [8]. In Egypt, where the agricultural sector faces the substantial challenge of producing more food with less water, the adoption of drip irrigation systems has proven to be a successful approach for a wide range of crops [9]. This technology plays a crucial role in achieving the dual goals of enhancing agricultural productivity and conserving water resources in an environmentally sustainable manner. On the other hand, sprinkler irrigation systems are characterized by their ability to provide uniform coverage over larger agricultural areas [10]. They are well-suited for applications in which widespread irrigation is required. Sprinklers can even serve the purpose of frost protection by creating a protective ice layer on crops during cold nights [11]. Furthermore, they typically have a lower upfront installation cost compared to drip systems, making them an attractive option in terms of initial expenditure.

The growing interest in harnessing natural resources underscores the significant importance of organic manure [12, 13]. The recycling of organic waste using sanitary methods is crucial for preserving soil health, ultimately contributing to higher crop yields and the well-being of humanity [14]. The inclusion of organic matter in the form of compost, farmyard manure, cereal residue, and green manure is recognized for its positive impact on the physical, chemical, and biological attributes of the soil [15]. According to Fischer and Glaser [16], composting stands out as the most pivotal and rewarding approach to enhance agricultural productivity by encouraging (a) the long-term structural stability, (b) the moisture retention capacity of the soil, and (c) nutrient availability. Furthermore, the composting of agricultural residues has the potential to mitigate global warming by reducing the reliance on agricultural chemicals and fertilizers, which, in turn, lowers the production of fertilizers and agricultural chemicals and leads to a decrease in greenhouse gas emissions [17]. Compost is a remarkable organic material that emerges from the natural decomposition of kitchen scraps, yard waste, and other biodegradable matter [18]. It offers a multitude of benefits for both gardening enthusiasts and the environment [18]. As a soil conditioner, compost enhances soil structure, moisture retention, and nutrient levels, fostering robust plant growth. It’s also a sustainable source of essential nutrients, releasing them gradually as it decomposes [19, 20]. The microorganisms present in compost bolster soil health by suppressing harmful pathogens and improving nutrient cycling [13]. Moreover, composting reduces organic waste in landfills, curbing methane emissions and contributing to sustainable waste management. Compost’s erosion control properties make it an ideal choice for landscaping, and its environmentally friendly nature promotes sustainability and cost savings in both gardening and agriculture [15]. Compost applications have emerged as a successful agricultural strategy, primarily due to their ability to enhance soil structure [21], increase water-holding capacity [22], boost soil nutrient availability [23], elevate soil enzyme activity, and improve soil physicochemical properties, even in demanding environmental conditions [24].

The utilization of potassium bicarbonate in wheat production can significantly enhance both crop yield and quality [25]. This compound serves a dual role in agriculture by providing essential potassium and regulating soil pH [26]. Adequate potassium levels are vital for promoting healthy wheat growth, including improved nutrient absorption and increased resilience to stress. Moreover, potassium bicarbonate’s fungicidal properties are beneficial for disease management, especially in controlling fungal threats like powdery mildew [27, 28]. By promoting a more balanced soil pH and mitigating such fungal attacks, potassium bicarbonate contributes to higher wheat yields and improved grain quality [25, 26, 29]. Nevertheless, the efficacy of this approach is contingent upon the specific soil conditions and management approaches employed, hence requiring the implementation of soil testing and the expertise of professionals to get the most favorable outcomes. Thus, the objectives of this study involve examining the impact of irrigation system, compost application method, and potassium bicarbonate on specific physiological characteristics, grain production, and components of wheat (cv. Giza-171) in newly reclaimed soil.

## Materials and methods

### Experimental site and experimental design and field management

Two field experiments were implemented during the winter seasons of 2020/21 and 2021/22 at the Experimental Farm of the Faculty of Agriculture, Al-Azhar University, Sadat City, Monofya Governorate, Egypt. This work was aimed to investigate the effect of irrigation system, compost application method and potassium bicarbonate on some physiological characters, grain yield, and its components of wheat plant (Variety Giza-171) under new reclaimed soil. The principal soil properties of the experimental site are presented in Tables (1 and 2).

**Table 1:** Physical properties of the upper 50 cm of the experimental soil site

Season	Fine sand %	Coarse sand %	Silt %	Clay %	CaCo_3_%	O.M %	Soil texture
2020/ 2021	38.40	35.00	15.07	8.23	2.45	0.94	Sand clay
2021/ 2022	35.95	36.50	13.95	6.63	6.35	0.63	

**Table 2:** Chemical properties of the upper 50 cm of the experimental soil site

Season	EC (mhos/cm)	pH	HCO_3_	Cl^−^	So_4_^−−^	Ca^++^	Mg^++^	Na^+^	K^+^	*N* (ppm)	*P* (ppm)	K (ppm)
2020/2021	3.53	7.23	7.19	12.28	11.46	7.03	6.45	14.84	0.68	28.34	14.46	234
2021/2022	2.08	6.49	6.67	10.45	6.75	6.18	4.12	11.28	0.45	21.38	10.27	241

The experimental design used was a split-split plot design with three replications. Irrigation system treatments were allocated in the main plots, compost application methods were distributed in subplots, and spraying with potassium bicarbonate treatment was randomly distributed in the sub-sup plots. Each experiment included 16 treatments which were the combinations between two irrigation systems (drip and sprinkler system) and four potassium bicarbonate spraying treatments: spraying rates, i.e., control (spraying with water), spraying with potassium bicarbonate 0.06 g/l, spraying with potassium bicarbonate 0.08 g/l and potassium bicarbonate 0.1 g/l. with two-way added compost under soil surface between 15 and 20 cm and mixed with soil. The experimental unit size was 10.5 m^2^ (3 m × 3.5 m). On 17 November 2020 and 20 November 2021, wheat grains (cv. Giza-171) were sown in lines, 10 cm distance, at a rate of 150 kg grains ha^− 1^. In this research, a common local wheat cultivar known for its high yield, named Giza-171, was used. The seed of Giza-171 cultivar was brought from Wheat Research Department, Field Crops Research Institute, Agricultural Research Center (ARC), Egypt. During land preparation, single super phosphate (15.5% P_2_O_5_), at a rate of 240 kg ha^− 1^, and gypsum, as a soil conditioner, at a rate of 2.4 t ha^− 1^, were incorporated. At 30 days after sowing (DAS), ammonium nitrates fertilizer (33.5% N) at a rate of 450 kg ha^− 1^ was applied.

This study employed two distinct irrigation systems: a drip irrigation system utilizing GR pipes with drippers spaced at 25 cm intervals along 3 meters in length, with a 20 cm spacing between lines; and a sprinkler irrigation system constructed using water sprinklers with a diameter of 3 meters. Potassium bicarbonate treatment was applied as foliar applications twice, 60 and 70 DAS. Four potassium bicarbonate substances treatments, (S_1_) spraying with water (Control), (S_2_) Spraying with potassium bicarbonate at the rate of 0.06 g / L, (S_3_) Spraying with potassium bicarbonate at the rate of 0.08 g / L and (S_4_) Spraying with potassium bicarbonate at the rate of 0.1 g / L. The compost was applied at a rate of 45 m3/ha and incorporated into the soil at a depth of 15–20 cm ‘Role method’ or mixed with the soil. The compost used in our study was commercially produced and is known as Nile compost. Nile compost is manufactured through pure aerobic composting of biomass and is characterized by its high organic material content and other fertilizing elements, including vitamins and natural antioxidants. Importantly, Nile compost is free of weed seeds, fungal pathogens, and bacteria, primarily nematodes.

### Measurements and calculations

Six plants were taken randomly from each plot 75 days after the sowing date. The plants were uprooted, and leaf area/plant (cm^2^) and plant highest were recorded, and leaf area index was calculated:

Leaf area index (LAI): It was calculated according to the following formula [30]:

$${\rm{LAI}}\,{\rm{ = }}\frac{{{\rm{Leaf}}\,{\rm{area}}\,{\rm{per}}\,{\rm{plant}}\,\left( {{\rm{c}}{{\rm{m}}^{\rm{2}}}} \right)}}{{{\rm{Ground}}\,{\rm{area}}\,\left( {{\rm{c}}{{\rm{m}}^{\rm{2}}}} \right)}}$$

To assess the Crop Growth Rate (CGR), plants with a length of 25 cm from each row were randomly selected at booting stage and the subsequent 20 days. The plants were then divided into roots and shoots and subsequently dried in a ventilated oven at 70 °C until a constant weight was obtained. The CGR parameter was calculated using the formula proposed by [30], defined as the rate of dry matter accumulation per unit of occupied ground per day (g /m-2/ week):

$${\rm{CGR}}\,{\rm{ = }}\left( {T2 - T1} \right)\,/\,(W2 - W1)$$

where W1 and W2 represent the dry weight of wheat plants at the booting stage and after 20 days, while T1 and T2 represent the time between the booting stage and the subsequent 20 days.

Net Assimilation Rate (NAR) is defined as the increased rate of plant dry matter per leaf area unit per time unit, as per [31]:

$${\rm{NAR = }}\,\left( {{{\rm{W}}_{\rm{2}}} - {{\rm{W}}_{\rm{1}}}} \right)\,{\rm{*}}\,\left( {{\rm{lo}}{{\rm{g}}_{\rm{e}}}{{\rm{A}}_{\rm{2}}} - {\rm{lo}}{{\rm{g}}_{\rm{e}}}{{\rm{A}}_{\rm{1}}}} \right)\,{\rm{/}}\,\left( {{{\rm{A}}_{\rm{2}}} - {{\rm{A}}_{\rm{1}}}} \right)\,{\rm{*}}\,\left( {{{\rm{W}}_{\rm{2}}} - {{\rm{W}}_{\rm{1}}}} \right)$$

Where W_1_, A_1_, W_2_, and A_2_, respectively refer to the dry weight and leaf area at times (T_1_ and T_2_) in weeks.

For grain yield and its components, at harvest (15th and 21st of April in 2020 and 2021 seasons, respectively), spike number m^− 2^ was measured. Moreover, ten plants were randomly obtained from each plot to measure thousand grain weight and grain number spike^− 1^. Finally, the whole plants of each plot were collected to estimate the grain yields ha^− 1^.

### Statistical analysis

Data of the two seasons was subjected to analysis of variance (ANOVA) according to [32]. SAS software program was used for carrying out ANOVA. Means were separated using Duncan’s multiple range test only when the F–test indicated significant (*p* ≤ *0.05*) differences among the treatments. The obtained data were subjected to analysis of variance analysis according to [33]. Differences among means were compared using LSD at a 5% probability level.

## Results

### Analysis of variance reveals significant impacts of agronomic inputs on wheat growth and productivity

To evaluate the influences of irrigation system, foliar potassium bicarbonate application, and composting method on wheat, ANOVA was conducted for the nine growth, physiology, and yield traits (Table 3). The analysis of variance showed that irrigation system (IS) had a significant effect on plant height (PH), leaf area index (LAI), crop growth rate (CGR), number of spikes per m^2^ (NSM), number of grains per spike (NGS), and grain yield (GY) in both years. The effect of IS on leaf area (LA) and thousand-grain weight (TGW) was only significant in 2020/21. Potassium bicarbonate rate (PBR) had a significant effect on all traits except TGW in 2020/21 and PH, LA, LAI, NAR, and CGR in 2021/22. The interaction between IS and PBR was significant for PH, LA, LAI, NSM, NGS, and GY in both years. compost application method (CM) significantly affected PH, LA, LAI, NSM, NGS, and GY in 2020/21 but only impacted NSM and NGS in 2021/22. The IS by CM interaction was significant for NSM and NGS in both years. The PBR by CM interaction was significant for PH, LA, LAI, and GY in 2020/21 but did not affect any traits in 2021/22. The 3-way interaction between IS, PBR, and CM was significant for PH, LA, and LAI in 2020/21 and for NGS in both years. This suggests a complex interaction between all three factors influencing certain wheat traits. Overall, irrigation system and potassium bicarbonate rate had the most consistent significant effects on wheat growth, physiology, and yield across the two years. The compost application method and its interactions with IS and PBR had significant but less consistent impacts over time.

**Table 3:** Analysis of variance for wheat growth, physiological and yield traits under two irrigation systems, four potassium bicarbonate rates, and two compost application method over two growing seasons (2020/21 and 2021/22)

Source	DF	Plant Height (cm)		Leaf Area (cm^2^)		Leaf Area Index		Net Assimilation Rate		Crop Growth Rate		No. Spikes/m^2^		No. Grains/Spike		Thousand grain weight (g)		Grain yield (t/ha^-1^)	
		2020/21	2021/22	2020/21	2021/22	2020/21	2021/22	2020/21	2021/22	2020/21	2021/22	2020/21	2021/22	2020/21	2021/22	2020/21	2021/22	2020/21	2021/22
rep	3	52.95	14.48	227.49**	228.94	0.06**	0.06	0.01**	0.00	0.19**	0.18	18.75	588.35	10.71	45.79	5.41	21.21	0.06	0.21
IS	1	805.14***	1521.00***	4257.56***	4025.90***	1.18***	1.12***	0.08***	0.21***	3.28***	8.24***	2025.00***	18496.00***	90.25***	72.25***	60.06***	210.25***	16.07*	28.69***
IS*rep	3	16.19	3.75	80.61	35.02	0.02	0.01	0.00	0.00	0.05	0.07	34.75	27.29	7.38	11.96	3.53	5.79	0.00	0.08
PBR	3	694.64***	573.19***	1938.08***	3292.42***	0.54***	0.91***	0.31***	0.99***	6.98***	28.11***	1547.33***	3001.69***	140.92***	179.58***	19.42**	74.25***	7.46	10.81***
IS*PBR	3	101.22**	119.88***	110.23	662.11***	0.03	0.18***	0.06	0.12***	1.10***	4.13***	27.00	93.88	8.92	11.58	5.23	6.92	1.10	1.89***
IS*PBR*rep	18	24.28	5.71	45.14	71.69	0.01	0.02	0.00	0.00	0.05	0.05	20.69	73.55	2.71	5.65	2.65	4.00	4.90	0.03
CM	1	293.27***	156.25***	1190.25***	1584.04***	0.33***	0.44***	0.00	0.04***	0.23**	1.62***	961.00***	1444.00***	72.25***	110.25***	36.00***	90.25***	1.58	1.52***
IS*CM	1	4.52	14.06	45.56	133.40	0.01	0.04	0.07***	0.01*	1.33***	0.07	0.00	105.06	12.25	2.25	3.06	0.25	0.00	0.01
CM*IS	3	6.43	1.38	16.08	46.68	0.00	0.01	0.05***	0.07***	0.88***	2.03***	29.67	24.04	6.92	6.92	2.50	4.25	0.07	0.05
IS*PBR*CM	3	4.27	20.10*	115.06*	98.13	0.03	0.03	0.03***	0.06***	0.46***	1.62***	11.33	31.85	2.92	2.92	1.06	4.92	0.07	0.19***

IS, irrigation system; PBR, potassium bicarbonate rate; CM, compost application method. *, **, *** indicate significant differences at *P* < 0.05, *P* < 0.01, and *P* < 0.001

### Effect of drip and sprinkler irrigation systems on wheat growth dynamics and yield components

**Fig. 1:**
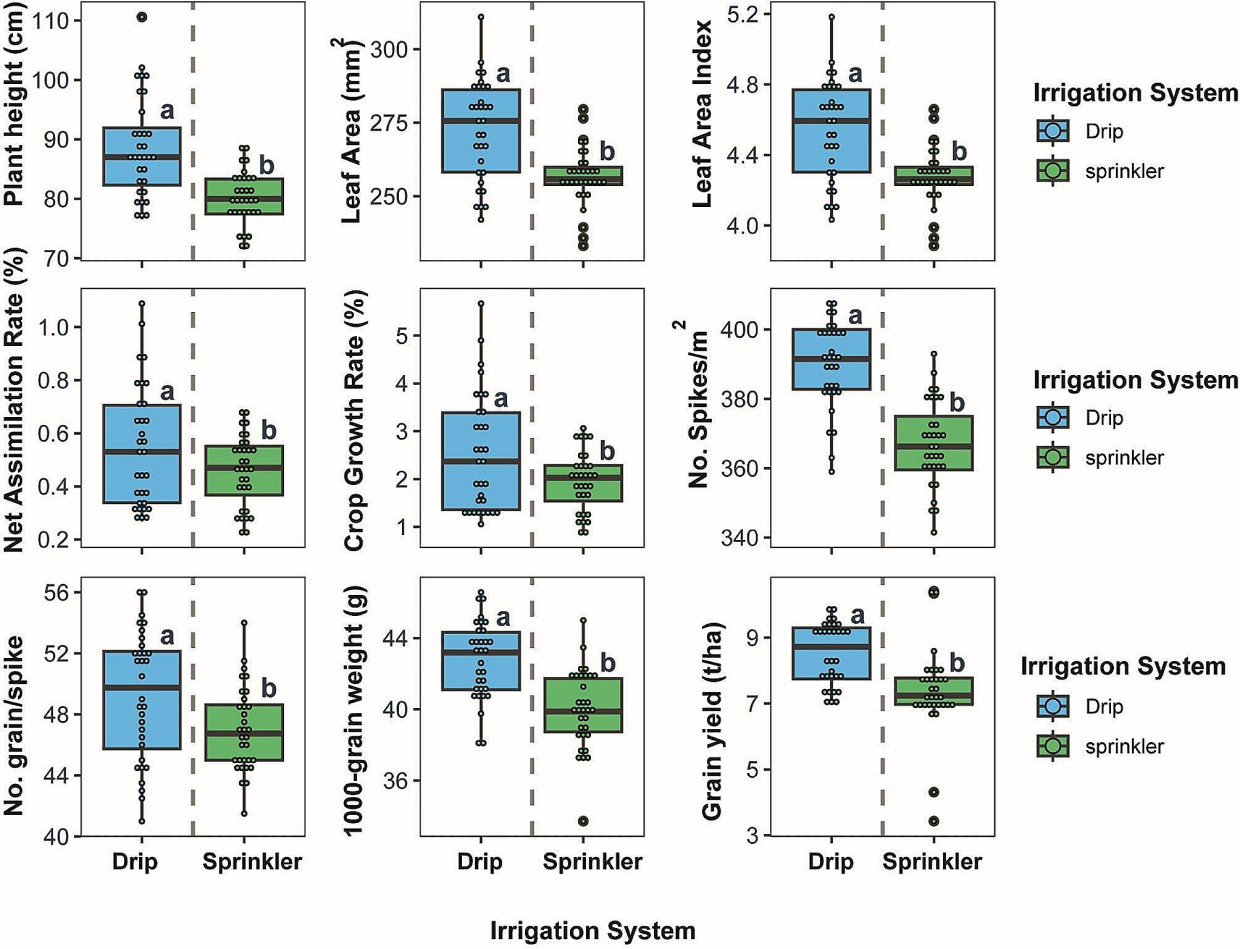
Boxplots exhibit differences in wheat growth and yield traits with drip irrigation compared to sprinkler irrigation. Central lines display the median, boxes represent the interquartile range, and whiskers indicate the total range excluding outliers. Letters in small cases on boxplots indicate different significant means of treatment at *P* < 0.05

The two irrigation systems imposed significant differences across all nine wheat traits assessed as shown in Fig. 1. Drip irrigation resulted in markedly higher values for each trait relative to sprinkler irrigation. Plant height increased by 10.6% under drip irrigation, averaging 88.59 cm versus 80.17 cm with sprinkler treatment. Leaf area showed a similar trend, averaging 272.56 cm^2^ with drip irrigation versus 256.47 cm^2^ for sprinklers, a 6.3% increase. The leaf area index followed suit, with drip irrigation resulting in a mean value of 4.54 compared to 4.27 with sprinklers, a 6.3% increase. Growth indices were substantially enhanced as well, with drip irrigation resulting in averages of 0.55 g/cm^2^/day for net assimilation rate and 2.54 g/cm^2^/day for crop growth rate, representing improvements of 19.6% and 30.3% over the sprinkler treatment means of 0.46 g/cm^2^/day and 1.95 g/cm^2^/day, respectively. Components of grain yield also showed considerable increases with drip irrigation, with mean values of 389.64 spikes/m2, 49.25 grains/spike, and 42.77 g for thousand grain weight under drip irrigation. This was 7.2%, 4.8%, and 7.0% higher than the sprinkler treatment means of 367.02 spikes/m^2^, 47.00 grains/spike, and 39.98 g, respectively. Notably, the coefficient of variation for net assimilation rate was 29.8% lower with drip irrigation compared to sprinklers, indicating more consistent growth. Ultimately, the collective improvements in growth and yield traits with drip irrigation culminated in a 16.0% increase in mean grain yield relative to sprinkler irrigation, averaging 8.52 and 7.35 g/m2 for the drip and sprinkler treatments respectively (Fig. 1).

### Optimizing foliar potassium bicarbonate application enhances wheat productivity

**Fig. 2:**
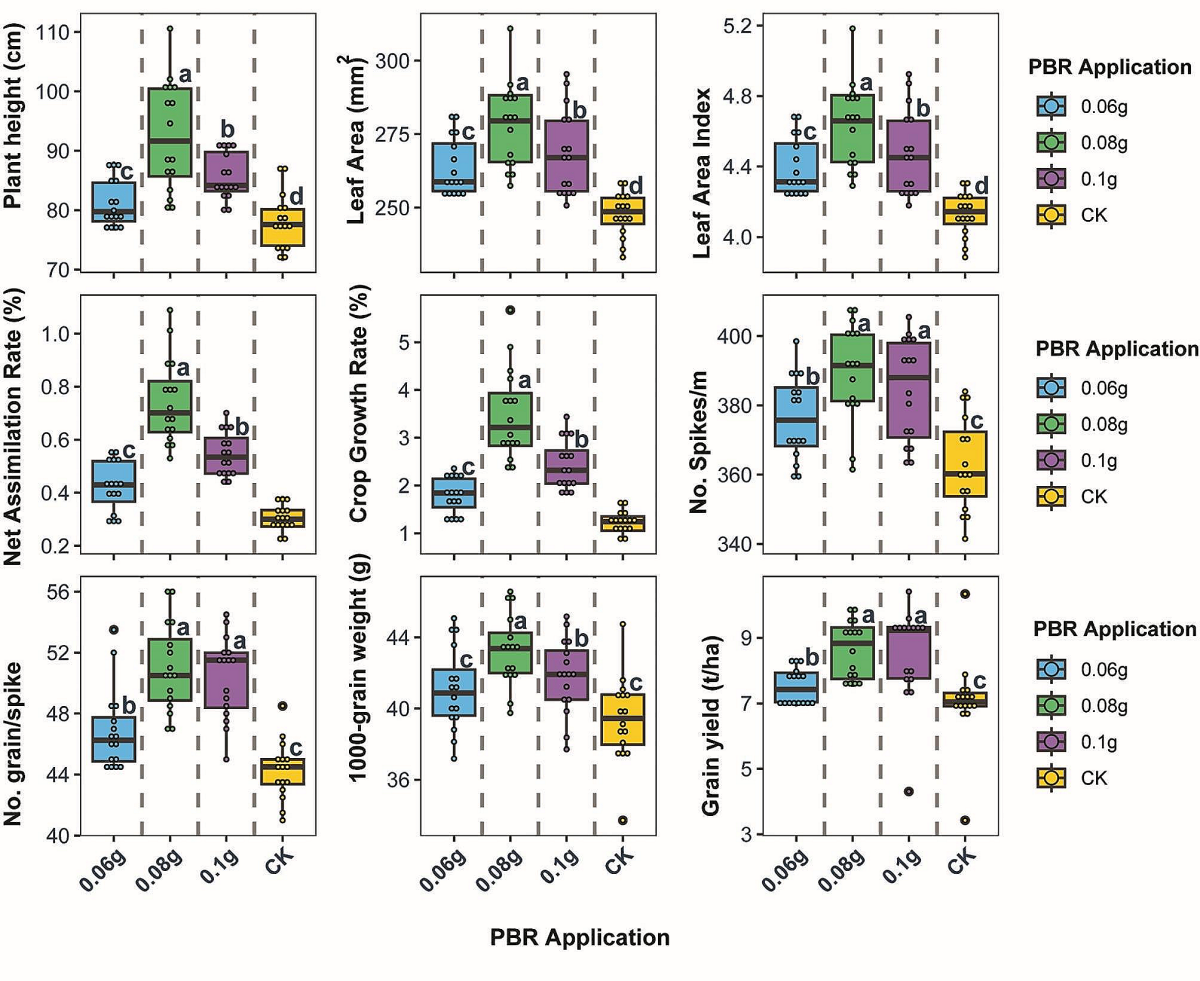
Boxplots show differences in wheat growth and yield traits among four levels of foliar potassium bicarbonate application. Central lines indicate median values, boxes represent the interquartile range, and whiskers show the total range excluding outliers. Letters in small cases on boxplots indicate different significant means of treatment at *P* < 0.05

Optimizing foliar potassium bicarbonate application enhances wheat productivity. Foliar application of PBR significantly influenced all nine wheat traits assessed in this study (Fig. 2). Plant height increased in a dose-dependent manner up to 0.08 g PBR, with 0.1, 0.08, and 0.06 g PBR resulting in 6.2%, 15.7%, and 3.9% taller plants compared to the unsprayed control, which averaged 78.06 cm. Leaf area and leaf area index showed similar trends, rising with increasing PBR up to 0.08 g, which maximized these traits at 12.3% and 12.8% over control means of 247.78 cm2 and 4.13, respectively. The 0.06 g PBR rate resulted in more modest 6.5% and 6.2% increases in leaf area and LAI versus the control. Growth rates displayed considerable improvements with PBR application. Net assimilation rate increased markedly from 0.31 g/cm2/day in the control to peak at 0.74 g/cm2/day in the 0.08 g PBR treatment, a 138% increase. Crop growth rate more than doubled from 1.24 g/cm2/day in control plants to a maximum of 3.49 g/cm2/day at 0.08 g PBR. Yield components also rose steadily up to 0.08 g PBR. This treatment maximized spikes/m2 at 389.62, representing a 7.4% increase over the control average of 362.84. Grains per spike peaked at 51.00 with 0.08 g PBR, 15.3% higher than the control mean of 44.25. Thousand-grain weight topped out at 43.25 g with 0.08 g PBR, 9.8% over the 39.38 g control average. Ultimately, grain yield showed stepwise increases with rising PBR up to 0.08 g, which maximized yield at 8.65 g/m2, 22% higher than the 7.09 g/m2 control average. This substantial yield enhancement with optimized PBR application demonstrates its strong potential to improve wheat productivity.

### Compost application method affects wheat growth dynamics and productivity

**Fig. 3:**
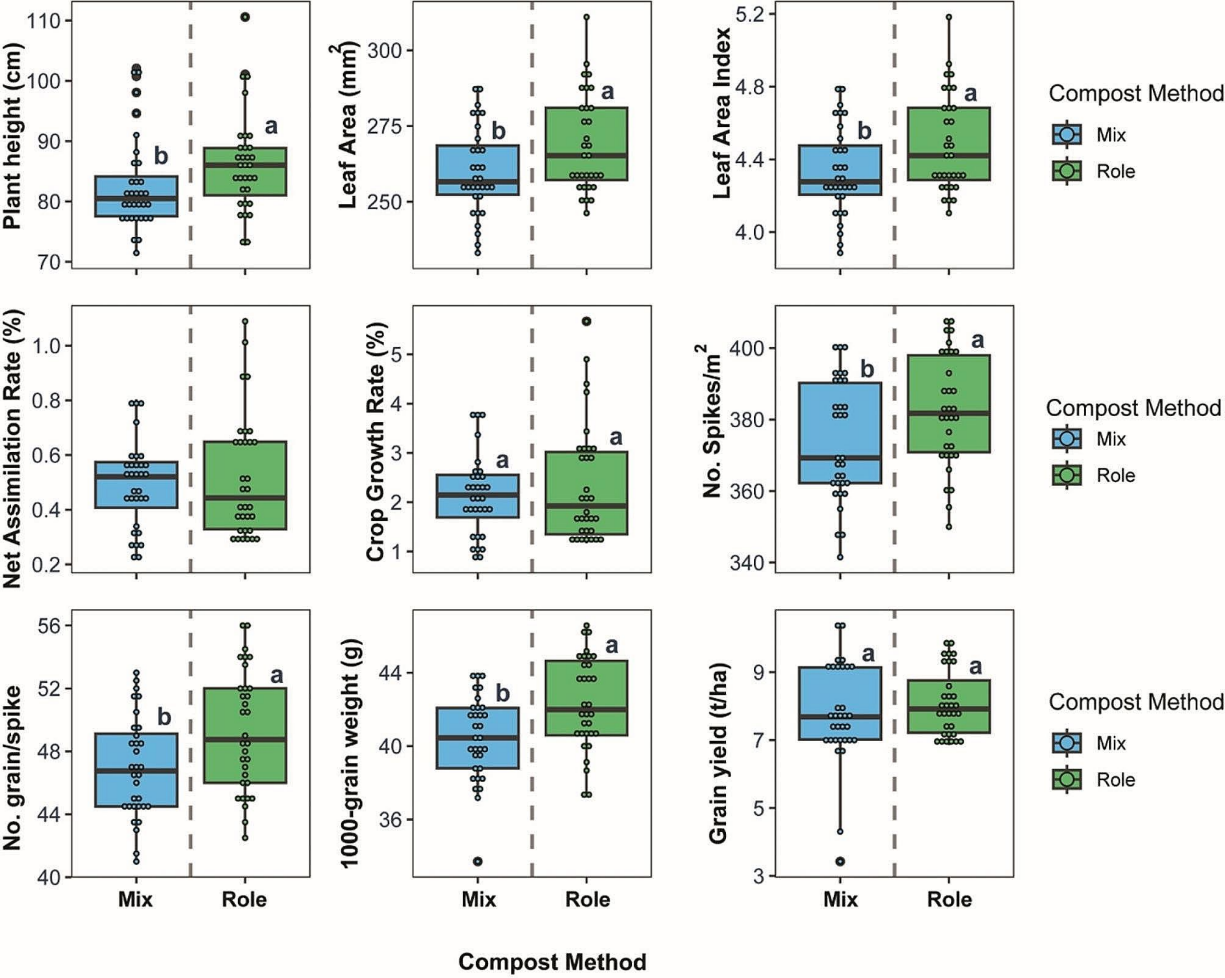
Compost application via the Role method enhanced wheat growth and yield versus Mix application. Central lines indicate median values, boxes represent the interquartile range, and whiskers show total range excluding outliers. Letters in small case on boxplots indicate differently significantly means of treatments at *P* < 0.05

The compost application methods imposed significant differences in wheat growth and yield traits. Compost applied via the Role method resulted in improved performance across all parameters versus the Mix application (Fig. 3). Plant height was 4.4% taller with Role, averaging 86.23 cm compared to 82.53 cm with Mix. The leaf area followed a similar trend, increasing by 3.5% with Role over Mix, with respective means of 269.16 cm^2^ and 259.87 cm^2^. The leaf area index also rose with Role, averaging 4.49 versus 4.33 for Mix, a 3.7% increase. Growth dynamics were enhanced with Role application as well. Net assimilation rate averaged 0.52 g/cm^2^/day with Role method versus 0.49 g/cm^2^/day with Mix, a 6.1% increase. Crop growth rate rose even more, up 10.3% with Role versus Mix, averaging 2.35 g/cm^2^/day and 2.13 g/cm^2^/day respectively. Grain yield components were improved with Role application too. Spikes per m^2^ increased by 2.3% with Role, averaging 382.64 versus 374.02 for Mix. Grains per spike were 5.2% higher with Role versus Mix, with respective means of 49.31 and 46.94. Role application improved thousand-grain weight by 4.8% over Mix, averaging 42.34 g versus 40.41 g. Ultimately, the collective improvements in growth and yield traits with Role compost culminated in 4.1% higher grain yield over Mix, with Role averaging 8.09 g/m^2^ compared to 7.78 g/m^2^ for Mix. The method of compost application significantly impacts wheat productivity.

### The effect of the three-way interaction on morphological, physiological, and yield traits

**Fig. 4:**
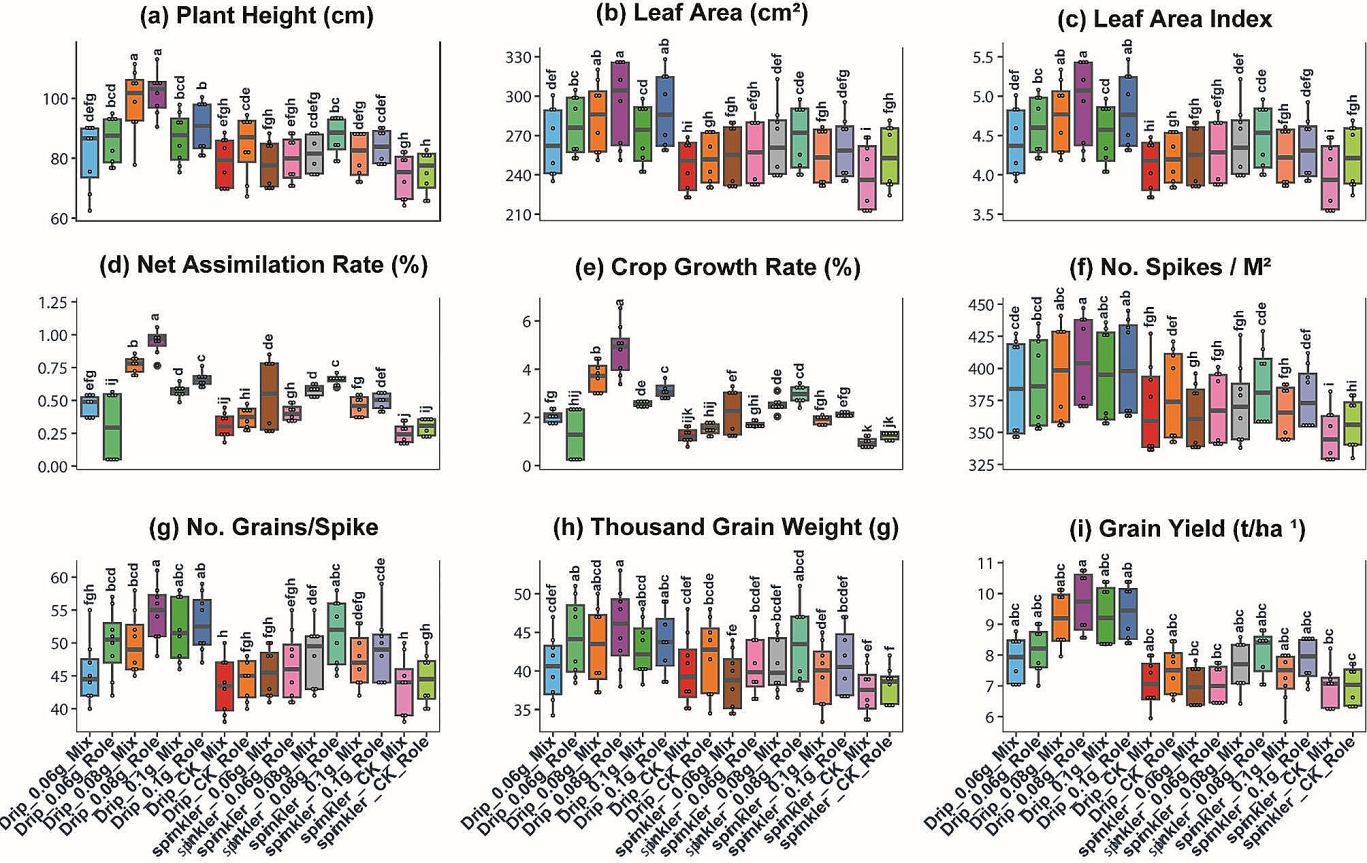
The effect of the triple interaction (irrigation system x potassium bicarbonate x composting method) on wheat growth and yield. Central lines indicate median values, boxes represent the interquartile range, and whiskers show total range excluding outliers. Letters in small case on boxplots indicate differently significantly means of treatments at *P* < 0.05

The combined analysis across the two seasons of study was assumed, and the three-way interaction between irrigation system, potassium bicarbonate rate, and compost application method is shown in Fig. 4. These results indicated that the effect of the three-way interaction had a significant effect on all nine traits related to wheat growth, physiology, and yield. Overall, the combination of drip irrigation, the potassium bicarbonate rate of 0.08 g, and the role composting method (Drip_0.08g_Role) resulted in the tallest plants, greatest leaf area and leaf area index, highest net assimilation rate and growth assimilation rate, highest no. of spikes, most grains per spike, heaviest thousand grain weight, and maximum grain yield. In contrast, sprinkle irrigation without potassium bicarbonate and with conventional mixed compost (sprinkle_CK_Mix) led to the poorest performance across all traits examined. It can also be noted that the drip irrigation system consistently outperformed sprinkle irrigation across the different potassium bicarbonate rates and compost application method in improving wheat growth, accelerating development, enhancing yield components, and increasing grain yield.

### Optimized agronomic inputs synergize to improve wheat productivity

**Fig. 5:**
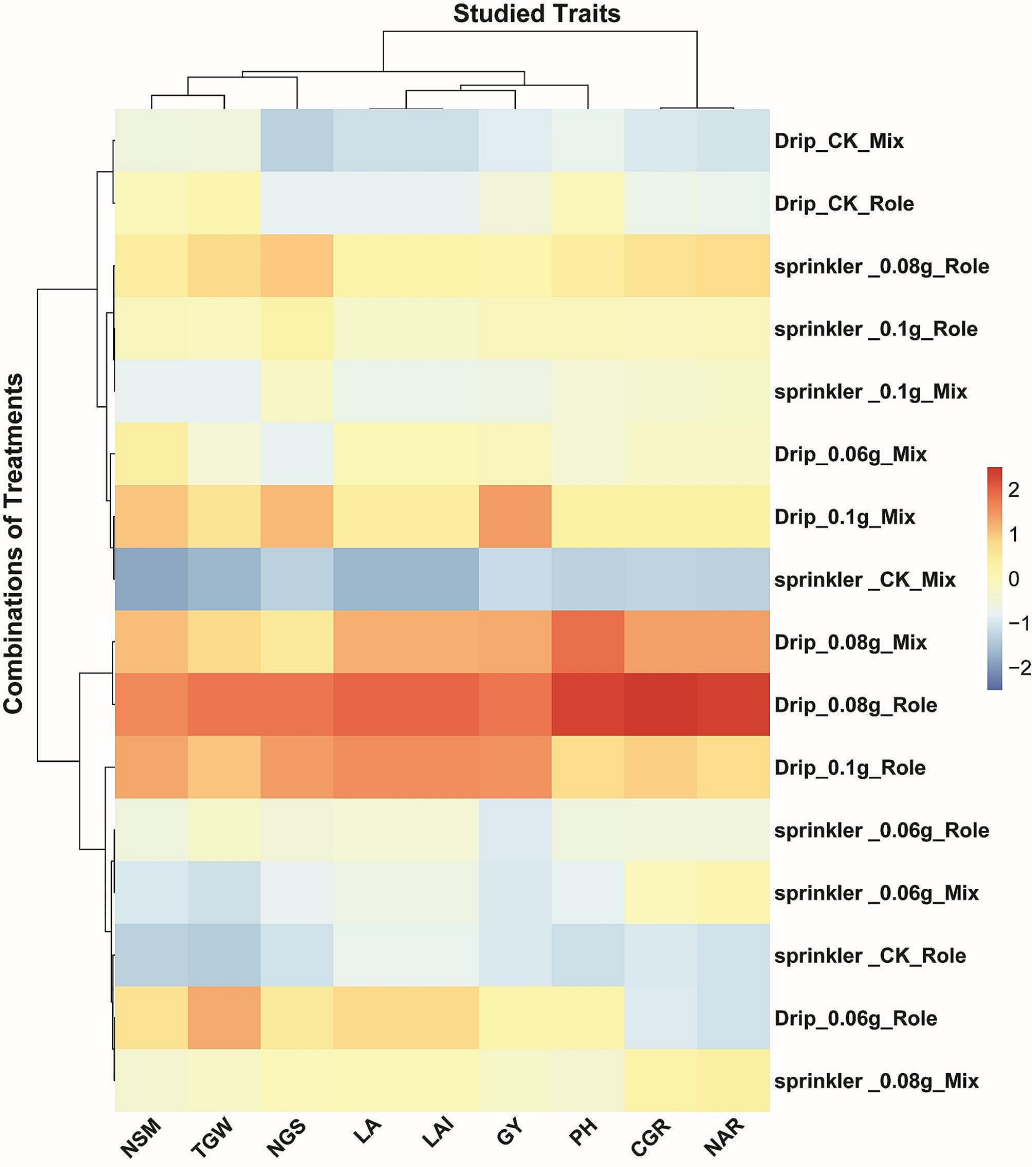
Heatmap of wheat trait responses to combinations of irrigation method, foliar potassium bicarbonate application, and composting technique. Red indicates positive, blue denotes negative effects on each trait. Notably, 0.08 g potassium bicarbonate application consistently improved productivity regardless of irrigation method or compost type. However, drip irrigation resulted in larger enhancements than sprinkler irrigation at a given nutrient rate. Overall, these findings demonstrate optimized agronomic inputs can synergize to substantially improve wheat growth, yield components, and productivity. PH, plant height; LA, leaf area; LAI, leaf area index; NAR, net assimilation rate; CGR, crop growth rate; NSM, no. spikes/m^2^, NGS, No. grains/spike, TGW, thousand grain weight; GY, grain yield

The combination of agricultural inputs can profoundly impact crop productivity. We assessed the effects of irrigation method, foliar potassium bicarbonate application, and composting technique on nine traits related to wheat growth and yield. Clustering of the trait heatmap indicates drip irrigation combined with 0.08 g potassium bicarbonate consistently optimized wheat performance across compost application method s, while sprinkler irrigation required higher nutrient rates to achieve comparable benefits (Fig. 5). Drip irrigation with 0.08 g potassium bicarbonate and Role compost application showed the most positive impacts across the traits, particularly increasing leaf area by 12%, leaf area index by 15%, net assimilation rate by 145%, crop growth rate by 32%, spikes/m^2^ by 9%, grains/spike by 8%, and ultimately grain yield by 18% compared to the control. Sprinkler irrigation with 0.08 g potassium bicarbonate and Role compost resulted in similar but slightly lower improvements in these traits. The positive effects of these treatments on growth dynamics highlight their importance in boosting grain yield. In contrast, the control drip and sprinkler treatments lacking supplemental nutrition had the most negative effects on the traits studied, reducing grain yield by 8–10%. This demonstrates the key role of nutritional supplements in optimizing wheat performance. Plant height and thousand grain weight were least affected by the input combinations. Notably, 0.08 g potassium bicarbonate application consistently improved productivity regardless of irrigation method or compost type. However, drip irrigation resulted in larger enhancements than sprinkler irrigation at a given nutrient rate. Overall, these findings demonstrate optimized agronomic inputs can synergize to substantially improve wheat growth, yield components, and productivity.

### Correlation analysis

**Fig. 6:**
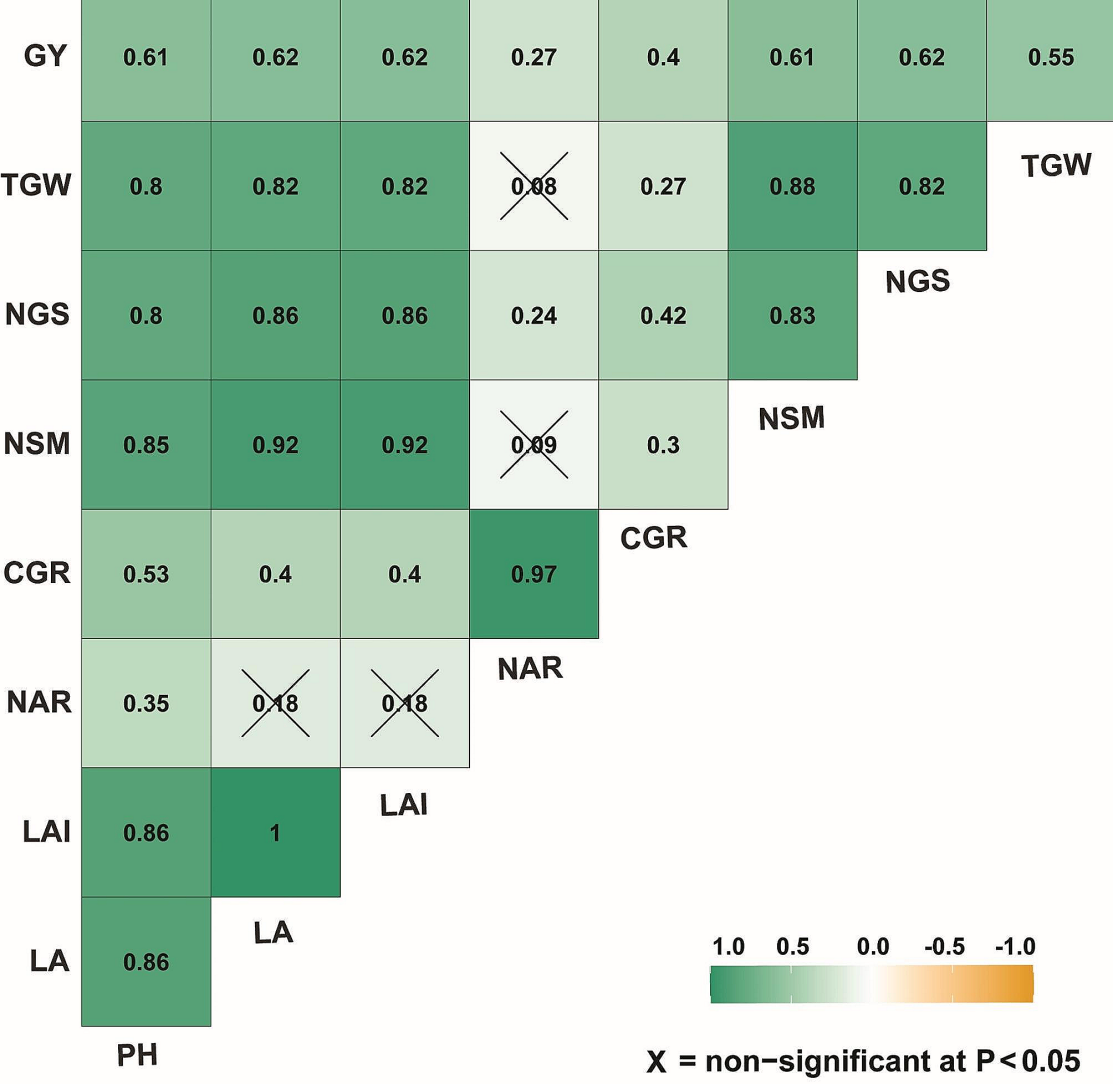
Correlation matrix showing associations between wheat growth and yield traits. PH, plant height; LA, leaf area; LAI, leaf area index; NAR, net assimilation rate; CGR, crop growth rate; NSM, spikes/m2; NGS, grains/spike; TGW, thousand-grain weight; GY, grain yield. Green-colored cells represent positive correlation coefficients, while orange-colored cells indicate negative correlation coefficients. The strength of the green color indicates a positive correlation, whereas the intensity of the orange color indicates a negative correlation. Crossed cells (with the sign X) indicate a non-significant coefficient of correlation

The correlation results provide insights into the relationships between the studied wheat traits, offering valuable information for wheat breeding and crop management strategies (Fig. 6). Plant height showed strong positive correlation coefficients with leaf area (*r* = 0.86), leaf area index (*r* = 0.86), crop gross rate (*r* = 0.53), number of spikes per square meter (*r* = 0.85), number of grains per spike (*r* = 0.80), thousand grain weight (*r* = 0.80), and grain yield (*r* = 0.61). A moderate positive correlation was observed with net assimilation rate (*r* = 0.35). For the physiological traits, leaf area exhibited strong positive correlations with leaf area index (*r* = 0.86), number of spikes per square meter (*r* = 0.92), number of grains per spike (*r* = 0.86), thousand grain weight (*r* = 0.82), and grain yield (*r* = 0.62). A moderate positive correlation was noted with crop gross rate (*r* = 0.40). The leaf area index displayed strong positive correlations with number of spikes per square meter (*r* = 0.92), number of grains per spike (*r* = 0.86), thousand grain weight (*r* = 0.82), and grain yield (*r* = 0.62). A moderate positive correlation was observed with crop gross rate (*r* = 0.40). Net assimilation rate showed weak positive correlations with crop gross rate (*r* = 0.97), number of spikes per square meter (*r* = 0.09), number of grains per spike (*r* = 0.30), thousand grain weight (*r* = 0.08), and grain yield (*r* = 0.27). Crop gross rate had moderate positive correlations with number of spikes per square meter (*r* = 0.30), number of grains per spike (*r* = 0.42), thousand grain weight (*r* = 0.27), and grain yield (*r* = 0.40). There was a moderate positive correlation with leaf area (*r* = 0.40). For the yield related traits, the number of spikes per square meter displayed strong positive correlations with number of grains per spike (*r* = 0.83), thousand grain weight (*r* = 0.88), and grain yield (*r* = 0.61). A moderate positive correlation was noted with the crop gross rate (*r* = 0.30). Number of grains per spike exhibited strong positive correlations thousand grain weight (*r* = 0.82), and grain yield (*r* = 0.62), while thousand grain weight showed strong positive correlations with grain yield (*r* = 0.55). The strong and moderate correlations identified among these traits can guide efforts to improve wheat varieties and optimize yield in agricultural practices.

## Discussion

Wheat constitutes a significant proportion of the global food supply. In light of soil erosion and climate change, it is imperative for farmers to explore novel and sustainable approaches to enhance wheat yields, thereby addressing the nutritional needs of growing populations. Our work adopts a comprehensive approach to optimize wheat crop output by examining the potential synergistic effects of irrigation methods, fertilizer inputs, and organic soil amendments. The implementation of a complete management approach is important in light of the complex interconnections between soil, water, and nutrients. Therefore, the primary objective of the present study is to enhance the resilience of wheat agriculture in the face of evolving environmental conditions [34, 35]. Furthermore, this study aims to illuminate the connections between food security issues. Hence, it is imperative to comprehend the impacts of irrigation systems, foliar potassium bicarbonate application, and composting techniques in order to enhance cultivation practices and enhance crop production for this essential crop. The study’s results indicate that drip irrigation is more effective than sprinkler irrigation in enhancing wheat development and yield outcomes, as it yielded significantly higher values in comparison to sprinkler irrigation. The observed improvements encompassed a 10.6% rise in plant height, a 6.3% increase in leaf area and leaf area index, a 19.6% increase in net absorption rate, and a 30.3% increase in crop growth rate. Drip irrigation significantly enhanced the yield components, resulting in a 7.2% increase in spikes per m2, a 4.8% increase in grains per spike, and a 7.0% increase in thousand-grain weight. Furthermore, it was observed that drip irrigation resulted in a higher level of growth consistency, as indicated by a decrease of 29.8% in the coefficient of variation for the net assimilation rate. The combined effects of drip irrigation on growth and yield resulted in a notable 16.0% rise in average grain yield compared to spray irrigation. This outcome is consistent with the conclusions found in numerous prior investigations [36, 37]. These findings underscore the importance of selecting an efficient irrigation system for wheat cultivation, particularly in regions where water resources are limited [10, 38]. Such superiority of drip irrigation can be attributed to its ability to provide water directly to the root zone and reduce water wastage can significantly enhance wheat productivity. In this regard, it has been reported that drip irrigation can optimize plant growth by positively modulating various physiological and biochemical processes, such as photosynthesis, respiration, translocation, ion uptake, carbohydrates, nutrient metabolism, and growth promoters [39–42].

Based on the results obtained from the present study, it was shown that the application of PBR had a notable positive impact on all evaluated wheat characteristics, exhibiting a dose-dependent relationship, with a dosage of 0.08 g. The optimized rate resulted in a 15.7% increase in plant height, a 12.3% increase in leaf area, a 12.8% rise in leaf area index, a 138% increase in net assimilation rate, and an 181% increase in crop growth rate compared to the unsprayed control. The yield components had a peak value of 0.08 g, characterized by an increase of 7.4% in spikes per square meter, 15.3% in grains per spike, and a 9.8% higher thousand-grain weight compared to the control group. In general, the application of PBR at a concentration of 0.08 g led to a 22% increase in grain yield compared to the control. The observed enhancements in wheat growth dynamics and yield components as the rate of foliar PBR administration increases up to 0.08 g demonstrate the potential of optimal foliar PBR application to significantly enhance wheat productivity, surpassing the performance of plants without supplementation. Research has provided evidence to support the notion that potassium (K) plays a pivotal role in the sustenance of photosynthesis and associated physiological mechanisms [25, 26, 28]. Zhao et al. [29] demonstrated that net photosynthetic rate of the uppermost fully expanded main-stem leaves of K-deficient cotton was only 23% of the control plants receiving a full K supply. Furthermore, potassium influences photosynthesis, which has a positive impact on vegetative characteristics [43, 44]. In other crops, such as potato, the rise in vegetative development sprayed with potassium sources could be attributed to enhancing assimilate translocation, protein synthesis, and enzyme activity promotion [43]. The increase in vegetative growth caused by spraying potato plants with potassium silicate could be attributed to potassium’s role in plant nutrition and enhancing assimilate and protein synthesis [45]. Also [27], outlined the importance of potassium as a nutrient for a number of physiological processes in plants, such as regulating gas and water exchange, protein synthesis, enzyme activation, and photosynthesis.

Regarding the application of compost, different methods could be responsible of significant influence on several traits, as revealed in this study. According to the results of this study, the composting role method generally outperformed the mix method. The efficiency of the role method of compost application was characterized by improved wheat growth and productivity compared to that mix method. Also, the role compost increased plant height by 4.4%, leaf area by 3.5%, leaf area index by 3.7%, net assimilation rate by 6.1%, and crop growth rate by 10.3% over the mix application method. Grain yield components also rose with role, including 2.3% more spikes per m2, 5.2% more grains per spike, and 4.8% higher thousand-grain weight versus mix method. Overall, these role compost positive influence was translated to a total increase of 4.1% in grain yield compared to the mix application. The results demonstrate that the method of compost application significantly impacts wheat performance, with the role placement conferring measurable benefits for growth and productivity over incorporating compost into the soil mix. Careful consideration of compost application strategy is warranted to maximize wheat outcomes. Importantly, adding organic residues to agricultural soils impacts enzyme activities involved in nutrient cycling [1, 46, 47]. Organic amendments have complex interactive effects on soil enzymes like phosphatase that regulate nutrient cycling [13, 48, 49]. Both the organic matter additions and sufficient nutrient inputs are needed to sustain the enzyme activity and mineralization processes [13, 48, 50, 51].

The cluster analysis conducted in this study revealed that the combination of drip irrigation and 0.08 g potassium bicarbonate resulted in the highest level of wheat performance across all compost types. On the other hand, sprinkler irrigation necessitated larger nutrient rates in order to achieve similar advantages. The application of drip irrigation, 0.08 g potassium bicarbonate, and Role compost resulted in the most significant improvements in leaf area, leaf area index, growth rates, yield components, and grain yield. These parameters experienced an increase of 8–18% compared to the control group. The application of sprinkler irrigation using the identical nutrition regimen yielded somewhat reduced however still significant enhancements in comparison to the control group. On the other hand, the drip and sprinkler control treatments that were not supplemented resulted in a decrease in production by 8–10%, underscoring the significance of nutritional inputs. The addition of 0.08 g of potassium bicarbonate consistently increased productivity, regardless of the irrigation mode or kind of compost used. However, drip irrigation showed greater benefits compared to sprinkler irrigation. The results of this study indicate that the implementation of optimum agronomic inputs, such as irrigation methods, foliar nutrition, and composting, can contribute to the enhancement of wheat growth dynamics, yield components, and productivity through a synergistic effect. The correlation analysis in this study showed that the strong positive correlation between plant height and leaf area and leaf area index indicates that taller plants tended to have larger leaves and more leaf area per unit ground area. Such findings were in line with those found in previous investigation [49, 52]. This makes sense biologically, as taller plants can support larger leaves and more leaf surface area. The moderate positive correlation between plant height and crop growth rate suggests that taller plants had higher growth rates, as the taller wheat plants likely had greater light interception and photosynthetic capacity to drive faster growth. In addition, the taller wheat plants seem to have higher spike and grain numbers and larger grains, all factors that would eventually contribute to higher grain yield [8, 54]. Collectively, this is likely due to the combined effects of greater light interception, photosynthesis, growth rates, and improvements in yield components. The strong positive correlation between leaf area and leaf area index is attributed to the fact that leaf area index is a measure of leaf area per unit ground area, so larger leaf area would directly translate to higher leaf area index. Additionally, the moderate positive of leaf area and leaf area index correlations with grain yield is also expected since larger leaf area leads to more photosynthesis to support growth and yield. Overall, by corroborating these biological relationships and consistency with earlier literature, the current correlation analysis provides greater confidence in the veracity of these linkages for wheat production. Furthermore, the integrated implications of the correlations suggest opportunities to optimize wheat yields through agronomic practices and genetics promoting taller, high-leaf area ideotypes with enhanced canopy photosynthesis and robust yield component expression.

## Conclusion

The present study offers significant contributions to the understanding of agronomic techniques in wheat cultivation, specifically focusing on the optimization of inputs and their effects on wheat growth and production. This inquiry highlights the crucial importance of irrigation systems, application of potassium bicarbonate, and composting techniques in improving the performance of wheat. Drip irrigation repeatedly shown superior performance compared to spray irrigation, leading to increased plant height, greater leaf areas, higher rates of growth, and a notable 16% increase in grain output. The foliar application of potassium bicarbonate, particularly at a dosage of 0.08 g, resulted in significant enhancements in wheat characteristics. This highlights the crucial involvement of potassium in many plant processes, ultimately resulting to a notable increase of 22% in grain output. Furthermore, the utilization of compost using the Role method showed inherent advantages over the Mix method, leading to a notable enhancement of 4.1% in grain yield. It is worth mentioning that certain combinations of these agricultural inputs showed synergistic effects, providing a comprehensive framework to improve the performance of wheat. These findings have significance for sustainable agriculture methods, not only for wheat cultivation but also for global food security and responsible resource management.

In conclusion, our study represents a significant step forward in the optimization of agrotechnical practices within wheat cultivation. However, there exists considerable potential for further exploration and refinement within this domain. To contextualize our findings within the broader landscape of agricultural research, future investigations could focus on evaluating the impact of alternative composts and bioproducts, particularly those augmented with beneficial microorganisms, on the physiological responses and yield outcomes of wheat plants under conditions of water deficit stress. Such inquiries hold promise for elucidating nuanced interactions between soil health, microbial communities, and plant performance, thereby contributing to the advancement of sustainable agronomic strategies tailored to the specific needs of wheat cultivation.

## Data Availability

The current study did not involve the generation of new sequencing data. Therefore, there are no datasets generated or analyzed during the current study.
